# Radical Nephroureterectomy Pentafecta as a Predictor of Upper Tract Urothelial Carcinoma Outcomes Following Radical Surgery

**DOI:** 10.1245/s10434-025-18411-5

**Published:** 2025-10-04

**Authors:** Chris Ho-Ming Wong, Ivan Ching-Ho Ko, David Ka-Wai Leung, Kang Liu, Hongda Zhao, Steven Leung, Pilar Laguna, Jean de la Rosette, Jeremy Yuen-Chun Teoh

**Affiliations:** 1 Department of Surgery, Faculty of Medicine, S.H. Ho Urology Centre, The Chinese University of Hong Kong, Hong Kong, China; 2 International School of Medicine, Istanbul Medipol University, Istanbul, Turkey; 3 Bashkir State Medical University, Ufa, Russia; 4 Li Ka Shing Institute of Health Sciences, The Chinese University of Hong Kong, Hong Kong, China; 5 Department of Urology, Medical University of Vienna, Vienna, Austria

**Keywords:** Upper tract urothelial carcinoma, Nephroureterectomy, Survival outcomes, Quality-of-care metrics, Pentafecta

## Abstract

**Introduction:**

Combined criteria have been used in many facets of urologic surgical care in the management of urological cancer. We aimed to validate the prognostic ability of a pentafecta related to the outcomes of radical nephroureterectomy (RNU) for upper tract urothelial carcinoma (UTUC).

**Patients and Methods:**

Data were obtained from the Clinical Research Office of the Endourology Society Urothelial Carcinomas of the Upper Tract (CROES-UTUC) registry, a prospective multinational database. Non-metastatic UTUC patients treated with RNU were included. We adopted a pentafecta criteria of (1) negative surgical margin; (2) en bloc resection of the bladder cuff; (3) absence of major complications; (4) template-based lymph node dissection performed per European Association of Urology guidelines; and (5) absence of recurrence (urothelial and/or distant recurrence) within 12 months. Outcomes were pentafecta achievement rates and oncological outcomes, including overall survival (OS) and recurrence-free survival (RFS). Kaplan–Meier survival analyses with log-rank were performed on survival outcomes. Multivariate Cox regression was performed to identify confounders, and logistic regression was performed to identify factors that confounded the pentafecta achievement rate.

**Results:**

Overall, 1049 cases were analyzed, and pentafecta was achieved in 504 patients (48.0%). Baseline characteristics were comparable between those who achieved pentafecta versus those who did not. Pentafecta achievement was associated with OS advantage (hazard ratio [HR] 0.586, *p *= 0.024) and RFS advantage (HR 0.291, *p *= 0.001). Multivariate Cox regression analysis identified that only pentafecta achievement and advanced T stage were independent predictors of RFS and OS. A ureteric location (compared with pelvicalyceal tumor) (odds ratio [OR] 0.424, *p *= 0.002), multifocality (OR 0.191, *p *< 0.001) and open RNU (OR 0.661, *p *= 0.010) were predictors of pentafecta non-achievement.

**Conclusion:**

We validated a pentafecta that gauged surgical quality for RNU. Quality-of-care metrics should be promoted to unify surgical outcomes in UTUC management.

Upper tract urothelial carcinoma (UTUC) is a relatively rare malignancy, representing only 5–10% of all urothelial tumors.^1^ Despite its lower incidence compared with bladder cancer, UTUC can be highly aggressive, with a substantial risk of early recurrences and disease-specific mortality.^2^

The European Association of Urology (EAU) guidelines recommend radical nephroureterectomy (RNU) as the gold-standard treatment for high-risk UTUC,^3^ which includes muscle-invasive or high-grade disease as identified by endoscopic or radiological evaluation. In a real-life clinical scenario, RNU is often the treatment of choice for low-risk UTUC patients who are good surgical candidates and have satisfactory contralateral kidney function.^4,5^ While RNU offers favorable oncological control for many patients, outcomes can vary significantly depending on tumor biology, surgical technique, and adherence to established perioperative measures such as bladder cuff resection and lymph node dissection (LND).^6^

In recent years, composite metrics or ‘fecta’ frameworks—combining oncological, surgical, and functional endpoints—have emerged as powerful tools to comprehensively assess the quality of urologic oncology procedures.^7^ For instance, pentafecta concepts have been successfully introduced in radical prostatectomy to integrate key performance indicators, including margin status, complications, continence, potency, and oncologic control.^8,9^ Similarly, early frameworks for partial nephrectomy incorporate parameters such as negative margins, minimal ischemia time, perioperative safety, and preserved renal function.^10–12^ These metrics offer a holistic snapshot of surgical achievement and help standardize reporting across centers, enabling meaningful comparisons that can drive quality improvement. However, until recently, no uniform composite endpoint existed to capture the critical elements of RNU, despite its recognized importance in UTUC management.

The present study addresses this gap by examining a pentafecta for RNU in patients with high-risk UTUC, encompassing five parameters: negative surgical margins, en bloc excision of the distal ureteral cuff, performance of LND in line with guidelines, freedom from major perioperative complications, and absence of any disease recurrence (urothelial and/or distant recurrence) at 12 months. Conceptual parallels have been drawn from recent work proposing tetrafecta or pentafecta endpoints for RNU.^13,14^ By applying this pentafecta to a large, multi-institutional cohort, we aimed to assess its feasibility, prevalence, and correlations with oncological outcomes such as recurrence-free survival (RFS) and overall survival (OS) in a real-life scenario of UTUC management. We believe that by examining such a composite measure, surgical proficiency and uniform quality reporting can be fostered in order to enhance patient care in UTUC.

## Patients and methods

### The Clinical Research Office of the Endourology Society Urothelial Carcinomas of the Upper Tract (CROES-UTUC) Registry

Data for the present analysis were sourced from The Clinical Research Office of the Endourology Society Urothelial Carcinomas of the Upper Tract (CROES-UTUC) registry.^15^ Established in 2014, this registry is one of the largest real-world, prospective, global databases in UTUC management, incorporating contributions from 29 participating centers across 101 countries. The registry is registered on ClinicalTrials.gov (NCT02281188)^16^ and adheres to the study protocol published according to the Agency for Healthcare Research and Quality guidelines for the design and use of patient registries for scientific, clinical, and health policy purposes.^15,17^ This study included consecutive patients over the age of 18 years diagnosed with non-metastatic UTUC and treated with RNU. Cases with known metastasis as prior operation, as well as those with insufficient baseline and/or follow-up data were excluded. Patient baseline characteristics, disease details, treatment information, and follow-up data were documented.

### Radical Nephroureterectomy Pentafecta

A surgical pentafecta of five different criteria was adopted, based on the pentafecta and surgical quality-of-care metric for RNU described by Konig et al.^13^ and Soria et al.^14^ These criteria were based on expert consensus and were proposed to reflect the quality of the overall management of UTUC. They covered three domains: surgical, postoperative, and oncological. Criterion 1, i.e. a negative surgical soft tissue margin and criterion 2, i.e. en bloc resection of the bladder cuff and criterion 3, i.e. LND performed per recommendation of the EAU guideline, covered the surgical domain. Criterion 4, i.e. absence of major complications within 30 days of operation, covered the postoperative domain, and criterion 5, i.e. absence of any disease recurrence within 12 months from operation, covered the oncological domain. Regarding the criterion of en bloc resection of the bladder cuff, this was defined as the adoption of open bladder cuff excision, or total laparoscopic RNU with en bloc resection of the bladder cuff, which was recommended by the EAU guideline. As related to the criterion of LND, the EAU guideline recommended that a template-based LND would be mandated for high-risk UTUC, while in low-risk UTUC, this would be optional. Regarding the criterion of absence of 12-month disease recurrence, recurrence was defined as recurrence at all sites, including local, contralateral kidney, bladder, or distant metastasis.

### Other Reported Parameters

Patient baseline characteristics, disease information, and treatment details were collected and analyzed. The reported tumor grading adhered to the World Health Organization classifications of 2004 and 2016. Complications were charted according to the Clavien–Dindo classification; grade 3 or higher complications were regarded as major. Disease staging was based on pathological examination of the RNU specimen in accordance with the TNM classification.^18^ Data collection was facilitated by the online Data Management System, a web-based platform located at the CROES office. Given the multicenter, multi-institutional, retrospective nature of this study, there was no standardized follow-up protocol, and hence the follow-up was performed in accordance with international guidelines in general.

### Definition of Outcomes

The analysis outcomes were (1) pentafecta achievement rates and (2) oncological outcomes, including RFS and OS. The cohort was then classified into the (1) pentafecta achievement group and (2) pentafecta non-achievement group for further analysis. RFS was defined as the absence of death or all-site recurrence (local, contralateral, distant and urothelial). Duration was defined as the period from operation until the occurrence of recurrence or death, while OS was defined as the interval from RNU to death. Patients were censored at the last follow-up.

### Statistical Analysis

The Pentafecta achievement rate was represented as the binary outcome. Differences in categorical variables between groups were assessed using Pearson’s Chi-square test or Fisher’s exact test as appropriate, while continuous variables were evaluated using the Mann–Whitney U test in a non-parametric fashion. Kaplan–Meier analysis with log-rank test was used to delineate RFS and OS. Univariate and multivariate Cox regression analyses (proportional hazards regression) were performed to identify potential confounders influencing RFS and OS. Variables included in the multivariate analysis were either those identified as significant (*p* < 0.05) or near-significant (*p* < 0.2) in the univariate analysis, known contributory factors documented in existing literature, or existed as baseline discrepancies between groups. Logistic regression was performed on the pentafecta achievement rate. All statistical tests were two-sided, with a *p*-value <0.05 considered statistically significant. Statistical analyses were conducted using SPSS version 25.0 (IBM Corporation, Armonk, NY, USA).

## Results

**Fig. 1:**
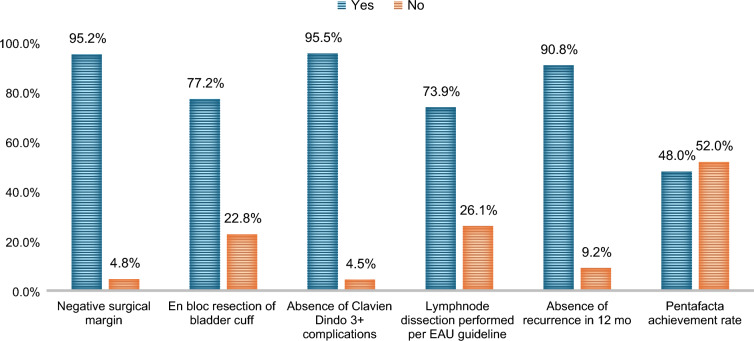
Pentafecta achievement rates. *EAU* European Association of Urology

Upon applying the inclusion and exclusion criteria, a total of 1049 patients were analyzed, Table 1 describes the pentafecta achievement rates of this cohort. Of the entire cohort, 999 patients achieved a negative surgical margin (95.2%); 810 patients received en bloc resection of the bladder cuff (77.2%). Absence of Clavien–Dindo grade 3 or higher complications was achieved in 1002 patients (95.5%), adherence to the EAU recommendation for LND was achieved in 775 patients (73.9%), and absence of recurrence was achieved in 953 patients (90.8%). Overall, 504 patients achieved all of these criteria (48.0%) (Fig. 1).

**Table 1:** Patient and disease characteristics according to pentafecta achievement

No. of patients, %	Pentafecta achievement		Pentafecta non-achievement		*p*-Value
	504	48.0%	545	52.0%	
Median follow-up duration, months	15.4		14.1		
Median age, years (IQR)	71	14	71	14	0.102
Sex (male:female), %	354:150	70.2%:29.8%	380:165	69.7%:30.3%	0.841
Median BMI, m^2^/kg (IQR)	25.2	5.2	25.4	5	0.138
Surgery duration, mins (SD)	193.5	90	180.4	86	0.069
Postoperative instillation, %	66	13.1%	59	10.8%	0.308
Length of hospital stay, days (IQR)	7	4	7	5	0.756
*Complications*					
All grade complications	80	15.9%	128	23.5%	0.031
Clavien–Dindo grade 1–2	80	15.9%	79	14.5%	
Clavien–Dindo grade 3 or higher	0	0%	39	7.2%	
Mean preoperative eGFR, mL/min/1.73m^2^ (SD)	63.4	22.7	63.3	22.8	0.916
Significant cardiovascular disease, %	61	13.3%	86	15.8%	0.323
History of smoking, %	279	55.3%	323	59.3%	0.128
ASA, %					<0.001
1 or 2	331	66.8%	308	56.5%	
3+	173	33.2%	237	43.5%	
Past history of, or concurrent, bladder cancer, %	66	13.1%	102	18.7%	0.013
Surgical approach, %					<0.001
Open	157	31.2%	222	40.7%	
Laparoscopic	287	56.9%	277	52.7%	
Robot-assisted laparoscopic	59	11.7%	46	8.4%	
Ipsilateral hydronephrosis, %	141	25.9%	172	31.6%	0.205
Tumor laterality (left:right), %	284:220	56.0%	278:267	51.0%	0.083
Tumor location,%					0.619
Ureter	110	21.8%	150	27.5%	
Renal pelvis	258	51.2%	254	46.6%	
Ureter and renal pelvis	57	11.3%	50	9.2%	
Missing	79	15.7%	91	16.7%	
Multifocality, %	86	17.1%	144	28.6%	<0.001
Histology, %					0.609
Grade 1	33	6.5%	35	6.4%	
Grade 2	117	23.2%	117	21.5%	
Grade 3	327	64.9%	362	66.4%	
Missing	27	5.4%	31	5.7%	
pT stage, %					0.815
Ta/is	9	1.8%	10	1.8%	
1	130	25.8%	145	26.6%	
2	133	26.4%	134	24.6%	
3	198	39.3%	227	50.8%	
4	34	6.7%	29	5.3%	

*eGFR* Estimated glomerular filtration rate, *pT* Pathological T stage, *IQR* Interquartile range, *BMI* Body mass index, *SD* Standard deviation, *ASA* American Society of Anesthesiologists

Upon classification to (1) pentafecta achievement and (2) non-achievement groups, the two groups had comparable patient and disease baseline characteristics (Table 1). Patient baseline characteristics, including age, body mass index (BMI), existing renal failure, and cardiovascular comorbidities, were comparable for the two groups. The pentafecta achievement cohort had a relatively larger portion patients with American Society of Anesthesiologists (ASA) grade 1–2 (66.8% vs. 56.5%, *p* < 0.001). For disease characteristics, the majority of tumors in both groups were located in the renal pelvis and calyces rather than the ureter (51.2% and 46.6%). Tumor grade, T stage, and the proportion of preoperative hydronephrosis of the operating side were comparable. The pentafecta achievement group consisted of more patients with multifocal tumors (17.1% vs. 28.6%, *p *< 0.001). With regard to treatment details, the pentafecta achievement group had more operations performed using a laparoscopic approach or robot-assisted laparoscopic approach than the non-achievement group (68.8% vs. 59.9%, *p *< 0.001). Postoperative stay, duration of surgery, and number of patients receiving intravesical chemotherapy after RNU were comparable (details are listed in Table 1). Upon assessing the potential factors related to pentafecta achievement using logistic regression, it was noted that multifocal tumor and ureteric tumor location were independent factors associated with pentafecta non-achievement, while the use of minimally invasive surgery (laparoscopic or robot-assisted laparoscopic) was a predictor of fecta achievement (Table 2).

**Table 2:** Multivariable logistic regression analyses for the prediction of pentafecta achievement

	Multivariate analysis			
		OR	95% CI	*p*-Value
Smoking history		0.945	0.677–1.319	0.738
Age		1.021	1.005–1.038	0.01
BMI		0.974	0.938–1.011	0.163
Female		1.051	0.73–1.514	0.788
*cT stage (Ta/is/1 as reference)*				
T2		0.882	0.577–1.349	0.563
T3		0.752	0.501–1.129	0.169
T4		1.17	0.525–2.609	0.701
Tumor grade (grade 1/2 as reference)		1.051	0.739–1.497	0.781
*Tumor location (Ref: pelvicalyceal)*				
Ureter		0.424	0.246–0.731	0.002
Ureteric and renal pelvic tumor		0.753	0.456–1.245	0.269
Multifocality		0.191	0.122–0.3	<0.001
Ipsilateral hydronephrosis		0.952	0.666–1.362	0.789
MIS RNU (open RNU as reference)		1.523	1.104–2.1	0.01
Previous bladder cancer		0.96	0.663–1.389	0.828

*BMI* body mass index, *MIS* minimally invasive surgery, *RNU* radical nephroureterectomy, *OR* odds ratio, *CI* confidence interval

**Fig. 2:**
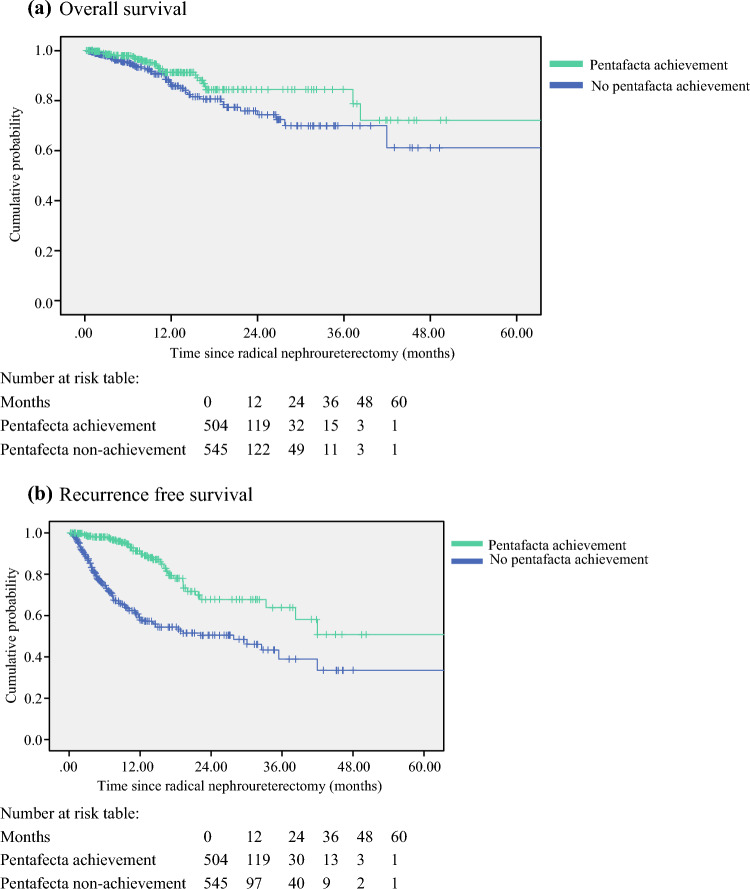
Kaplan–Meier estimates of oncological outcomes according to pentafecta achievement. **a** Overall survival; **b** recurrence-free survival.

The pentafecta achievement group was associated with superior OS (hazard ratio [HR] 0.586, 95% confidence interval [CI] 0.357–0.962, *p *= 0.035) and RFS (HR 0.291, 95% CI 0.203–0.419, *p *< 0.001) compared with the non-achievement group. The Kaplan–Meier survival plots of OS and RFS are presented in Fig. 2. In the univariate analysis for factors contributing to OS, pentafecta non-achievement, multifocality, advanced tumor T stage, tumor grading, and the use of adjuvant systemic chemotherapy were predictors of inferior survival (Table 3a). Upon multivariate analysis, pentafecta non-achievement was the only significant factor (HR 0.549, 95% CI 0.315–0.956, *p *= 0.034). In the multivariate regression analysis for RFS, pentafecta non-achievement (HR 0.248, 95% CI 0.167–0.367, *p *< 0.001), advanced T stage (HR 2.321, 95% CI 1.412–3.817, *p *= 0.0041) and adjuvant chemotherapy (HR 3.127, 95% CI 2.023–4.835, *p *< 0.001) were statistically significant factors for inferior outcomes (Table 3b).

**Table 3:** Univariable and multivariable Cox regression analyses for the prediction of oncological outcomes

	Univariate analysis				Multivariate analysis			
	Effect size	95% CI		*p*-Value	Effect size	95% CI		*p*-Value
*(a) Overall survival*								
Pentafecta achievement	0.586	0.357	0.962	0.035	0.549	0.315	0.956	0.034
Smoking history	1.268	0.776	2.073	0.343	–	–	–	–
Age	1.015	0.993	1.038	0.188	–	–	–	–
Sex (male as reference)	1.428	0.825	2.473	0.203	–	–	–	–
Existing chronic kidney disease	1.311	0.813	2.116	0.267	–	–	–	–
Past history of bladder cancer	1.196	0.699	2.048	0.514	–	–	–	–
Multifocality	1.682	0.954	2.963	0.072	1.285	0.7	2.36	0.418
Surgical approach (open as reference)								
Laparoscopic	1.014	0.598	1.718	0.96	–	–	–	–
Robot-assisted laparoscopic	1.219	0.58	2.564	0.602	–	–	–	–
pT stage (Ta/is as reference)								
T2	2.02	0.847	4.821	0.113	1.397	0.545	3.582	0.486
T3	3.59	1.682	7.659	0.001	2.287	0.964	5.421	0.06
T4	6.179	2.136	17.875	0.001	3.853	1.173	12.657	0.026
Tumor grade (grade 1 and 2 as reference)	2.838	1.404	5.737	0.004	1.751	0.821	3.737	0.147
Adjuvant chemotherapy	2.656	1.471	4.796	0.001	1.853	0.945	3.636	0.073
Adjuvant bladder instillation	0.869	0.415	1.818	0.709	–	–	–	–
*(b) Recurrence-free survival*								
Pentafecta achievement	0.291	0.203	0.419	<0.001	0.248	0.167	0.367	<0.001
Smoking history	1.3	0.946	1.786	0.106	1.126	0.783	1.619	0.522
Age	1.01	0.996	1.025	0.176	–	–	–	–
Sex (male as reference)	1.315	0.931	1.858	0.12	1.297	0.871	1.933	0.201
Existing chronic kidney disease	1.388	1.022	1.886	0.036	1.127	0.8	1.588	0.495
Past history of bladder cancer	0.99	0.692	1.418	0.958	–	–	–	–
Multifocality	1.456	0.995	2.132	0.053	0.894	0.602	1.33	0.582
Surgical approach (open as reference)								
Laparoscopic	1.088	0.771	1.536	0.63	–	–	–	–
Robot-assisted laparoscopic	1.263	0.783	2.036	0.338	–	–	–	–
pT stage (Ta/is as reference)								
T2	1.657	0.985	2.786	0.057	1.305	0.75	2.272	0.347
T3	2.849	1.817	4.466	<0.001	2.321	1.412	3.817	0.001
T4	5.62	2.979	10.601	<0.001	4.939	2.482	9.825	<0.001
Tumor grade (grade 1 and 2 as reference)	2.318	1.521	3.532	0	–	–	–	–
Adjuvant chemotherapy	2.765	1.883	4.061	<0.001	3.127	2.023	4.835	<0.001
Adjuvant bladder instillation	0.762	0.461	1.26	0.29	–	–	–	–

*CI* confidence interval

## Discussion

RNU remains the gold-standard treatment for high-risk UTUC,^3^ however surgical outcomes and long-term oncologic benefits still vary widely depending on operative technique, institutional expertise, and patient selection; in particular, the adoption of minimally invasive surgery is growing.^19,20^ In recent years, the introduction and validation of composite measures—termed ‘fecta’ metrics—have allowed clinicians to quantify and compare treatment quality and post-treatment outcomes across different procedures in urologic oncology.^21^ For example, the concept of pentafecta has been increasingly studied and adopted in radical prostatectomy and partial nephrectomy, helping to consolidate functional, oncologic, and perioperative parameters into a single composite endpoint.

Evidence for RNU has remained scarce until recently, when multi-institutional collaborations have put forth standardized criteria to define RNU fecta.^13,14^ In the present study, we adopted a pentafecta framework that examines key surgical, perioperative, and early oncologic endpoints for RNU. While the achievement rates of each of these metrics ranged from 70% and higher, we found that approximately half of our cohort met these five criteria. This highlights that operative considerations (e.g., surgical margin status, bladder cuff technique, thorough LND), perioperative safety (i.e., avoidance of major morbidity), and early oncologic control each contribute considerably to quality-of-care metrics. The significant associations between pentafecta achievement and oncological outcomes were in parallel with earlier data in which composite metrics were shown to predict long-term outcomes in RNU.^13,14^ The consistency between our findings and these multi-institutional cohorts underscores the pivotal role that technical excellence and judicious perioperative management play in UTUC care. Indeed, negative soft-tissue surgical margins reduce tumor seeding and local spread,^22^ while adequate bladder cuff management mitigates the risk of ureteral stump recurrence, with incidence rates as high as 58% in incomplete resection scenarios.^23,24^ Likewise, routine or template-based LND, particularly in high-risk or muscle-invasive UTUC, aids in accurate staging, and defines better indications for adjuvant therapies.^25,26^

Our data further highlight the importance of perioperative safety measures in driving outcomes, with the absence of major complications correlating with improved survival. This observation mirrors experiences in RNU as well as in other urologic surgeries,^27,28^ where composite endpoints consistently include avoidance of high-grade complications, confirming the powerful negative effect that severe morbidity exerts on patient recovery and survival. The relatively high proportion of patients attaining the ‘no major complications’ criterion demonstrates that even in a real-world scenario level with diverse surgical approaches, it is feasible to maintain low morbidity when established safety protocols and surgical skill sets are in place.

We additionally demonstrate that the ‘no early recurrence’ criterion serves as a surrogate marker of both technical proficiency, appropriate patient selection, and adequate perioperative systemic therapy. In fact, including an oncological criterion among other treatment-associated factors was more controversial but was not unheard of.^29^ The absence of early recurrence criterion is a surrogate marker for multiple crucial steps in UTUC management, such as correct case selection,^30^ adequate perioperative systemic treatment,^31^ and/or intravesical instillation.^32,33^ Including a criterion of oncological outcomes alongside functional outcomes was first proposed in a cohort of radical prostatectomy^29^ and was adopted in a multitude of urological oncology surgeries^34–36^ (Table 4). Its use in UTUC management stemmed from the different facets in the perioperative care of RNU. While being a relatively complex and aggressive cancer with a multitude of perioperative interventions accumulating in updated evidence, such as neoadjuvant and/or adjuvant systemic therapy (chemotherapy and immune checkpoint inhibitors), as well as neoadjuvant and/or bladder instillation, to capture all of these factors in one fecta set of criteria. A composite surrogate such as this would hopefully underscore the above factors, all of which must be harmonized to avert early recurrence. Using a 12-month timepoint as a cut-off of ‘early’ recurrence stemmed from the tumor biology of UTUC. It is known that UTUC is associated with high propensity of early recurrence, with all-site recurrence risks following RNU being as high as 47%.^37–39^ Therefore, a 12-month period would be able to capture a substantial number of at-risk postoperative patients.

**Table 4:** Summary table illustrating urologic oncology procedures utilizing fecta-based assessment tools for outcomes prediction

Study (year)	Procedure and approach	Disease details	Setting	‘Fecta’ components	Pentafecta rate (%)	Key predictors of ‘fecta’ achievement
Patel et al. ^36^	RARP	Localized prostate cancer (mostly D’Amico low risk)	Single-surgeon series (>1000 RARPs)	(1) Urinary continence (0 pads) (2) Sexual potency (intercourse-capable erections) (3) Biochemical recurrence-free (PSA ≤0.2 ng/mL) (4) No significant perioperative complications 5) Negative surgical margins	70.8	Younger age
Hung et al. ^10^	Robotic or laparoscopic PN (with hilar clamping during the entire course of the operation, early unclamping and zero ischemia techniques)	Patients with renal masses (mostly T1a disease)	Single-surgeon series	(1) Negative tumor margin (2) Minimal renal function decline (3) No urological complications	44–68	Not mentioned
Cacciamani et al. ^35^	Robot-assisted RC + ICUD with intracorporeal ileal conduit or neobladder and ePLND	Bladder cancer (muscle-invasive and high-risk non-muscle invasive)	Single-institution	(4) Negative soft-tissue surgical margins (5) ≥16 lymph nodes removed 6) No major complications (Clavien–Dindo III–V) within 90 days (7) No diversion-related sequelae ≤12 months (8) No clinical recurrence ≤12 months	53.3	Younger age Ileal-conduit diversion pN0
Current study	RNU	Non-metastatic UTUC treated with open or minimally invasive RNU	Multcenter	(1) Negative surgical margin (2) En bloc resection of bladder-cuff (3) Absence of major complications (4) Lymph node dissection performed per EAU guidelines (5) Absence of recurrence within 12 months	48.0	Renal pelvic location Unifocal disease Minimally invasive RNU

*RNU* Radical nephroureterectomy, *RARP* Robot-assisted laparoscopic radical prostatectomy, *PN* Partial nephrectomy, *PSA* Prostate-specific antigen, *ICUD* Intracorporeal urinary diversion, *UTUC* Upper tract urothelial carcinoma, *EAU* European Association of Urology

Nevertheless, it is important to recognize certain limitations. First, as with many multi-institutional datasets, differences in surgeon experience, technique standardization, and follow-up protocols might introduce heterogeneity. The data analysis was retrospective in nature. Selection bias, variations in center and surgeon experience, and heterogeneity of a center’s follow-up protocol could not be accounted for. The limitation of follow-up duration may also impose bias into the analysis, as late recurrences and metastasis might not be captured adequately. While we aimed to address this through consistent definitions and data management**,** future validation in prospective trials or meticulously designed registries would strengthen our findings and potentially refine the pentafecta criteria, especially knowing that fecta criteria might perform differently in different healthcare settings and patient populations. Second, the role and extent of LND, while part of our pentafecta definition, remains somewhat debated. Although guidelines recommend template-based LND in muscle-invasive UTUC, real-world adoption varies and a definitive consensus across risk strata has yet to be fully established. Furthermore, the lack of uniformity in the LND template could not be adequately solved as no single template is recommended by the guidelines as yet. Moreover, our study represents one of the early attempts to validate the clinical use of RNU fecta. We acknowledge that data on quality of life, functional outcomes, and patient-reported outcomes (that were addressed in prostatectomy and nephrectomy fectas) are generally lacking, for example renal function decline and its subsequent impact on general health. While the analysis is a retrospective examination of prospectively collected data, limitations on data have to be addressed**,** such as data multicollinearity that could be present in the multivariable regression analysis, and the lack of potentially important variables (e.g. neoadjuvant and adjuvant systemic therapy regimens), despite our careful attempts to select variables that could be adopted for the regression analysis. Finally, our limited follow-up duration may not capture late events. While a 12-month event-free interval is a suitable proxy for acute and early–mid oncologic risks, later recurrences could also be influenced by the quality of the initial surgery.

Looking ahead to the everyday clinical scenario, pentafecta or its variants offer a unifying metric for auditing, benchmarking, and continuous quality improvement in UTUC management, providing benefits that single endpoints alone cannot achieve. At the research level, these surgical quality-of-care metrics can serve as comparative benchmarks during the exploration of novel UTUC therapeutic tactics, such as robotic surgery, segment resection, or intraluminal instillation of chemotherapeutic agents.^40^

## Conclusion

We validated a pentafecta that gauged surgical quality for RNU, which can be recommended for the standardization of UTUC care. Quality-of-care metrics should be further promoted to unify and improve surgical outcomes in the surgical management of UTUC.
